# What has changed in the prevalence of hypertension in dialyzed children during the last decade?

**DOI:** 10.1080/0886022X.2016.1260033

**Published:** 2016-11-24

**Authors:** Marcin Tkaczyk, Małgorzata Stańczyk, Monika Miklaszewska, Katarzyna Zachwieja, Ryszard Wierciński, Roman Stankiewicz, Agnieszka Firszt-Adamczyk, Jacek Zachwieja, Halina Borzęcka, Ilona Zagożdżon, Beata Leszczyńska, Anna Medyńska, Piotr Adamczyk, Maria Szczepańska, Wojciech Fendler

**Affiliations:** a Department of Pediatrics, Immunology and Nephrology, Polish Mother’s Memorial Hospital Research Institute, Lodz, Poland;; b IV Chair of Pediatrics, Medical University of Lodz, Lodz, Poland;; c Department of Pediatric Nephrology, Jagiellonian University Medical College, Krakow, Poland;; d Department of Pediatrics and Nephrology, Medical University of Bialystok, Bialystok, Poland;; e Department of Pediatric Nephrology, Torun, Poland;; f Department of Cardiology and Nephrology, Medical University of Poznan, Poznan, Poland;; g Department of Pediatric Nephrology, Medical University of Lublin, Lublin, Poland;; h Department of Nephrology and Hypertension of Children and Adolescents, Medical University of Gdansk, Gdansk, Poland;; i Department of Pediatric Nephrology, Medical University of Warsaw, Warsaw, Poland;; j Department of Pediatric Nephrology, Medical University of Wroclaw, Wroclaw, Poland;; k Department of Pediatric Nephrology, Silesian Medical University, Zabrze, Poland;; l Department of Pediatrics, Hematology, Oncology and Diabetology, Medical University of Lodz, Lodz, Poland

**Keywords:** Dialysis, hypertension, pediatrics, prevalence

## Abstract

**Background:** Hypertension very often accompanies progression of chronic kidney disease (CKD) in children. A cross-sectional analysis of hypertension prevalence in dialyzed children in Poland was designed with a comparison with the data previously recorded 10 years earlier.

**Methods:** Two cohorts of children were analyzed: 59 subjects dialyzed in 2013, and 134 children from the previous study performed in 2003 that were reevaluated according to the current methodology. The incidence of hypertension (defined by SDS of sBP or dBP >1.64), clinical data, medical history, dialysis modalities and selected biochemical parameters of dialysis adequacy were analyzed.

**Results:** The prevalence of hypertension increased from 64% in 2003 to 78% in 2013. The efficacy of antihypertensive treatment remained unsatisfactory (61% proper BP control). Preservation of residual urine output and strict fluid balance may prevent development of hypertension in children on dialysis.

**Conclusions:** Despite the higher awareness of hypertension and its complications in dialyzed children, the incidence of this entity has increased during the last decade, with the percentage of undertreated patients comparable to that observed 10 years ago. Thus, more attention should be paid to therapy efficacy in this population to prevent further damage to the cardiovascular system and to decrease morbidity.

## Introduction

Hypertension frequently accompanies progression of chronic kidney disease (CKD) both in children and in adults. Usually, the percentage of hypertensive patients increases along with the progression of CKD from stage 1 to 5. In the cohorts of dialyzed children, regardless of the populations studied, all the authors reported the values over 70%.^1–3^ Undoubtedly, the problem of hypertension in dialyzed children is at least as high as in adult population.^4^ Young adults suffering from CKD since early childhood are in high-risk group of death due to cardiovascular complications.^5^^,^^6^

The activation of the renin–angiotensin–aldosterone system and sodium retention play a pivotal role in renal hypertension regardless of the stage of the disease.^7^ Arterial stiffness in uremia significantly increases also in children and uremic endothelial damage additionally supports its development. Furthermore, drugs commonly administered in CKD can cause iatrogenic hypertension (e.g., erythropoietin, glicocorticosteroids, growth hormone).^4^^,^^7^

In numerous studies, it has been ascertained that special attention should be paid to adequate diagnosis and treatment of hypertensive dialyzed children. At least for 10 years, nephrologists have been aware of a low effectiveness of high blood pressure treatment in this population.^2^^,^^3^^,^^8^^,^^9^ However, the cardiovascular surveillance is still not adequate.^10^ It is also well known that for adequate BP control, the majority of dialysis patients need a combination of several antihypertensive drugs.^11^

To evaluate the changes in hypertension prevalence and the therapeutic approach to hypertension in chronically dialyzed children (on peritoneal dialysis—PD, or hemodialysis—HD) that could have happened within the last 10 years, a cross-sectional analysis in all pediatric dialysis units in Poland was designed.^1^

## Methods

The study was designed as a cross-sectional analysis of hypertension prevalence among the Polish population of chronically dialyzed children in the period of April to June 2013. All the 11 pediatric dialysis centers were approached with a request to collect data. Ten centers responded. The historical data of 134 dialyzed children from the study performed in 2003 were reassessed and combined with these recently obtained.^1^

### Study group

The inclusion criteria encompassed age (0–18 years) and chronic dialysis (HD or PD) for at least 3 months. The study group consisted of 59 chronically dialyzed children (aged 3–222 months). Detailed characteristics of this group are presented in Table 1.

**Table 1.** Clinical characteristics of the study group (2013) and recalculated data from 2003.

	2013			2003	
	All children *N* = 59	Hypertensive children *N* = 46	Normotensive children *N* = 13	Hypertensive children *N* = 86	Normotensive children *N* = 48
Age (months)	128 (61–171)	132 (78–189)	99 (38–158)	141 (91–193)	120 (53–160)
M:F ratio	2.1:1	1.6:1	12:1^b^	2.2:1	1.5:1
PD:HD ratio	37:22/1.7:1	29:17/1.7:1	8:5/1.6:1	1.8:1	2.2:1
Duration of dialysis (months)	16 (6–27)	16 (7–27)	14 (6–21)	17.5 (6–36)	20 (11–42)
Duration of CKD (y.)	6 (4–11)	6 (2–10)	4 (1–12)	7 (3–18)	5 (1–13)
Microangiopathy (%)	7/59 (12%)	7/46 (15%)	0/13 (0%)	16/86 (19%)	3/48 (6.2)
Cardiomyopathy (%)	9/59 (15%)	8/46 (17%)	1/13 (8%)	18/86 (21%)	6/48 (12%)
Positive family history for hypertension	11/59 (19%)	8/46 (17%)	3/13 (23%)	16/86 (16%)	8/48 (18%)
Epoietin dose^a^	121.2 (70.7–160)	131 (83.6–156.8)	89.5 (55.5–181.8)	100 (72–170)	86 (49–173)
Urinary output (ml/kg/d)	17 (1.6–38.0)	12.5 (2.7–31.7)	26 (0.0–40)	9.7 (0–21)	35 (56–158)
Height (cm)	125 (102–149)	129 (102–154)	115 (91–145)	137 (117–144)	132 (105–146)
Height SDS	−2.2 (−3.4 to −1.0)	−2.4 (−3.5 to −1.0)	−2.1 (−2.7 to −1.4)	−2.5 (−3.6 to −1.2)	−2.2 (−3 to 1.5)
Weight (kg)	24 (17–40)	28 (15–41)	22 (12–36)	34 (20–42)	31 (15–49)
Weight SDS	−1.3 (−1.9 to −0.7)	−1.5 (−1.9 to −0.7)	−1.1 (−1.5 to −0.9)	−1.5 (−2 to −0,6)	−1.2 (−1.5 to −0.8)
BMI (kg/m^2^)	16 (15–19)	17 (14–19)	16 (15–18)	16 (14.3–18.0)	16.2 (15.3–17.0)
BMI SDS	−0.5 (−1.0 to 0.0)	−0.5 (−1.1 to −0.1)	−0.3 (−0.7 to 0.2)	−0.5 (−1.0 to 0.5)	−0.4 (−0.7 to 0.3)

Data presented as median value and 25–75 interquartile range.

BMI: body mass index; CKD: chronic kidney disease; HD: hemodialysis; PD: peritoneal dialysis.

a calculated for epoietin beta.

b significantly different *p* <0.05.

### Data collected for analysis

The study form included the following records: CKD data, dialysis modality, its efficacy, anthropometrics, blood pressure values, additional clinical data (i.e., family history, urine output, antihypertensives and other drugs that might interfere with blood pressure), as well as selected laboratory parameters (local laboratory: hemoglobin, KT/V, serum protein and albumin, vitamin D_3_). A specific clinical form to be filled out (by treating physician) for every patient included into the study was designed. The data were sent back to coordinating center, checked for consistence and analyzed.

Blood pressure (systolic: SBP, diastolic: DBP) measurements were performed employing a standard procedure applied for every clinical center (different oscillometric devices). Blood pressure during the routine PD clinical visit and a mean of three consecutive measurements (in a week) just before the hemodialysis procedure (predialysis) were recorded.

Blood pressure measurements were compared to the normative values of the Polish population and the standard deviation score (SDS, Z-score) was calculated.^12^ For children younger than 5 years of age, normative data were derived from the 4th Report.^13^ Hypertension was diagnosed for BP equal or higher than 95th percentile (1.64 SDS) for systolic BP or diastolic BP. Adequate control of hypertension was achieved when the treated patient’s BP was below 95th percentile (1.64 SDS).

Patients’ height was assessed employing standard methods. For the purpose of the analysis, in case of HD children, posthemodialysis weight was recorded, whereas morning weight (calculated without dialysis fluid) was determined for PD children. Anthropometric measurements were compared to the Polish national growth charts.^14^^,^^15^ Assessment of retinopathy was done by trained ophthalmologist, whereas echocardiography by trained cardiologist (presence of LVH).

Statistical analysis. The results are expressed as medians and 25th–75th percentiles. Statistical comparisons between the groups were made by the two-sided unpaired *t*-test or Mann–Whitney test. For multiple group comparisons, the authors used the nonparametric analysis of variance (Kruskal–Wallis test). If analysis of variance (ANOVA) yielded significant results, *post hoc* intergroup comparisons were performed using the Bonferroni–Dunn test. The Spearman’s rank correlation coefficient was used to evaluate associations between continuous variables. The chi-square and Fisher exact test were applied to compare the categorical variables. Multivariate analysis (in pooled groups) was performed using general linear regression model which estimated the effect of hypertension, time and confounding clinical variables. *p* values lower than 0.05 were considered significant.

### Findings

#### Prevalence of hypertension

In the current cohort of patients, 38/59 were diagnosed as hypertensive by the managing physician and treated, whereas 8/59 patients had abnormal high blood pressure values (diastolic or/and systolic) and were left untreated. Thus, the final hypertension prevalence was 78%, being higher than the reevaluated (with new growth and blood pressure charts) data originating from 2003, where the reevaluated prevalence rate was 64% (86/134) (*p* = 0.04). The aggregate (2003–2013) hypertension rate in the dialyzed children in Poland equaled 68%.

There was no significant difference in the prevalence of high blood pressure between HD and PD children, or between male and female subjects. Younger children (below 5 years) demonstrated the same rate of hypertension as older patients.

#### Adequacy and treatment complications

The percentage of patients with BP within the normal range (below 95th percentile) among hypertensive children was assessed. It was found that 22/38 children were undertreated and eight underdiagnosed (treating physician did not report hypertension), leading to an overall number of 30/46 (65%) children treated inadequately. After comparing these observations with the 2003 cohort, no significant increase in the treatment efficacy (65% in 2003) was found. After analysis of the influence of age on treatment efficacy, we revealed that children below and over 5 years of age revealed a similar prevalence of hypertension and a comparable rate of successful treatment as older ones.

Clinical complications of hypertension and CKD included microangiopathy (assessed by standard ophtalmoscopy) and cardiomyopathy (i.e., left ventricle hypertrophy: LVH). Seven (15%) hypertensive children were positive for the former complication, whereas eight (17%) demonstrated the latter. Only one normotensive child presented with cardiovascular complications (Table 1).

### Hypertension risk factors

#### Clinical factors

**Figure 1.**
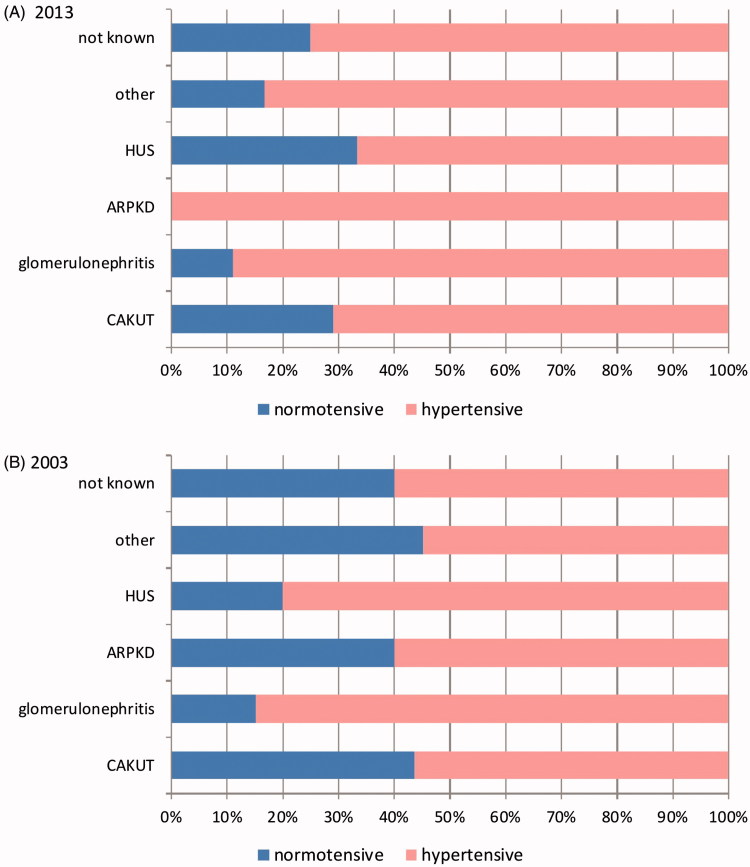
Proportion of hypertensive to normotensive patients in the study group according to the diagnosis of CKD.

Two groups of children were compared with respect to the clinical factors that proved their significance in the previous studies. The distribution of underlying diagnosis of CKD (CAKUT/glomerulonephritis/other) among hypertensives and normotensives was similar; likewise was the PD/HD ratio (Table 1). Figure 1 presents the proportion of hypertension in various clinical CKD entities. The presence of glomerulonephritis, ADPKD and hemolytic uremic syndrome as an underlying cause presumably resulted in a higher incidence of hypertension. It was confirmed by the analysis in the pooled cohort, where a higher incidence of glomerulonephritis in hypertensives was encountered (32% vs. 13% in normotensives, *p* = 0.0196). Nevertheless, in both cohorts, CAKUT was the most frequent diagnosis.

#### Biochemical parameters

Based on the previous studies, a comparison between selected biochemical parameters in normotensive and hypertensive children was performed, first in the cohort of 2013, then for the pooled cohort of 2003–2013. The authors intended to find potential factors associated with systolic and diastolic blood pressure (Tables 2 and 3, respectively). In 2013, the observed disparities were not significant, but the tendency towards lower hemoglobin concentration values in hypertensives was noted. In the pooled data (used for the multiple regression analysis), it was clearly visible that hypertensive children demonstrated lower residual urinary output and lower daily net fluid removal (including PD/HD ultrafiltration).

**Table 2.** Biochemical values in the study group (2013).

	All children *N* = 59	Hypertensive children *N* = 46	Normotensive children *N* = 13	Statistical difference (*p*)
KT/V	2.1 (1.5–2.5)	2.0 (1.5–2.5)	2.1 (1.4–2.8)	ns
Serum total protein (g/L)	67 (61–70)	67 (62–70)	64 (60–69)	n
Hemoglobin(g/dL)	10.7 (9.9–11.7)	10.3 (9.6–11.5)	11.3 (10.7–12.6)	0.08
Serum Ca (mmol/L)	2.4 (2.3–2.6)	2.4 (2.3–2.6)	2.4 (2.3–2.6)	n
Serum P (mmol/L)	1.9 (1.7–2.2)	1.9 (1.7–2.2)	2.0 (1.75–2.1)	n
Serum vitamin D (25-OH)	20.6 (16.3–29.6)	20.2 (16.2–29.4)	22.8 (18.5–33.2)	n
Serum albumin (g/L)	38 (35–43)	39 (36–43)	37 (31–41)	n

Data presented as median value and 25–75 interquartile range.

**Table 3.** Comparison of selected clinical and biochemical variables between hypertensive and normotensive patients in the pooled cohorts (2003–2013).

	Normotensives *n* = 61 (13 + 48)			Hypertensives *n* = 132 (46 + 86)			
	Median value	25th centile	75th centile	Median value	25th centile	75th centile	*p*
Age (months)	124	57	162	144	81	192	0.052
Length of renal replacement therapy (months)	20	11	40	16	7	28	ns
Z-score of height	−2.09	−3.25	−1.48	−2.19	−3.48	−1.04	ns
Z-score of weight	−1.28	−2.00	−0.70	−1.48	−2.10	−0.81	ns
Z-score of body mass index	−0.43	−0.90	0.11	−0.53	−1.11	−0.12	ns
Urine output (mL/kg/day)	33	3.8	61.5	11.2	1.2	27.6	0.002
Net fluid removal (UF + diuresis) (mL/kg/d)	57.1	38.1	79.9	39.0	27.9	61.9	0.001
KT/V	2.10	1.40	2.80	2.04	1.5	2.52	ns
Serum total protein (g/d)	65	61	68	64	58	69	ns
Hemoglobin (g/dL)	11.1	10.2	11.8	10.9	10.0	11.7	ns
Serum phosphate (mmol/L)	2.00	1.75	2.13	1.88	1.68	2.20	ns
Serum parathormone (pg/mL)	279	234	698	385	155	762	ns
Serum calcium (mmol/L)	2.40	2.27	2.60	2.41	2.28	2.56	ns
Serum uric acid (mg/dL)	6.50	5.50	8.00	6.00	5.40	7.10	ns
Serum albumin (g/L)	37	31	41	39	36	43	ns
Epoietin dose (IU/kg/week)^a^	89	58	163	111	56	172	ns

a calculated for epoietin beta.

### Correlation analysis

Clinical and biochemical factors were included into the correlation analysis. Systolic BP SDS correlated negatively with daily urine output (*R*= −0.16, *p*= 0.0319), length of CKD (*R*= −0.30, *p*< 0.0001), total protein (*R*= −0.21, *p*= 0.0034) and hemoglobin (*R*= −0.16, *p* = 0.0304). Diastolic BP correlated with age at CKD diagnosis (*R*= 0.18, *p*= 0.0134) and daily diuresis (*R*= −0.22, *p*= 0.0025). The analysis showed that diastolic blood pressure differed significantly depending on the underlying diagnosis (*p*= 0.0057 Kruskal–Wallis ANOVA). Patients with glomerulonephritis as the cause of CKD had higher diastolic blood pressure when compared to CAKUT (*p* =0.0064) and patients with other causes of CKD (*p* = 0.0372).

The multivariate analysis (within the pooled group of 2003–2013 children) proved the significance of duration of CKD (β= −0.19; *p* = 0.0179) and urinary output (β= −0.22; *p* = 0.0046) for systolic blood pressure SDS and urinary output (β = −0.18; *p* = 0.0475) for diastolic blood pressure SDS. Duration of CKD was associated with diastolic blood pressure, but this relation did not reach statistical significance (β= −0.15; *p* = 0.0587).

## Discussion

In the present analysis, it was demonstrated that the incidence of hypertension in the dialyzed children in Poland increased within the last decade. In 2003, it was assessed (after reevaluation according to Polish charts) as 64%, whereas in 2013 as 78%. The increase was an unexpected phenomenon, because of major increase in clinical knowledge about dialyzed children hypertension. The authors expected to find an increase rather than a decrease of therapy adequacy.^2^ However, in keeping with the recently published studies, similar tendencies are observed in the United States and Europe.^16^ Chavers et al. reported the 63% incidence of hypertension in a large cohort of hemodialyzed children,^17^ whereas in another study, the incidence was 79%.^2^ In another analysis performed in a smaller number of patients, the incidence was slightly lower (59%).^18^

In a large European registry, abnormal blood pressure was significantly more prevalent in: (1) cohorts of very young patients (under 3 years of age) as compared to 13- to 17-year olds (odds ratio (OR): 2.47); (2) during the first year compared to an over 5-year period of renal replacement therapy (RRT) duration (OR: 1.80); and (3) in patients on hemodialysis compared to transplant recipients or those on peritoneal dialysis (ORs: 2.48 and 1.59, respectively).^16^

The authors faced a comparable (65%) rate of children treated inadequately in 2003 and 2013. Chavers et al. reported in 2009 that the 74% of hypertensive hemodialyzed children were undertreated in the United States.^2^ Among subjects receiving antihypertensive treatment, uncontrolled BP was associated with male sex, shorter chronic kidney disease duration and the absence of angiotensin-converting enzyme inhibitor or angiotensin receptor blocker use.^19^ Taking into account those reports, the inadequacy of the hypertension treatment might be a significant burden. Halbach et al. also indicated the significance and timeliness of this problem in CKD children, likewise Flynn et al.^19^^,^^20^

The numbers of failures in hypertension treatment between 2003–2013 were comparable. The explanation of this phenomenon is not simple, but it can be hypothesized that in the cohort of 2013, the clinical condition of the dialyzed children was more severe as compared to the former group (selection bias). On the other hand, it may be assumed that the awareness of necessity for standard hypertension diagnosis and treatment among Polish nephrologists did not increase. This may lead to suboptimal preventive cardiovascular care that may worsen cardiovascular mortality in young adults in the near future.^10^

The lifespan of children on dialysis in the United States is 40–60 years shorter and for transplant patients, about 20–25 years shorter than that of an age- and race-matched US population.^21^ The most likely cause of this phenomenon is an increased cardiac mortality that could be affected by the presence of hypertension.^22^ The detrimental impact of these complications on mortality is relatively higher in pediatric population than in adults, despite the fact that mortality rates decreased significantly over the last decade.^9^^,^^23^ Bakkaloglu et al. assessed LVH as highly prevalent; furthermore, they demonstrated that low BP and renal dysplasia (as a CKD cause) were protective against this complication.^24^ The frequency of cardiac complications in the current study was assessed by echocardiography. In Poland, screening of all the dialyzed children for LVH or cardiomyopathy is a standard procedure. In a single-center Polish study, 13/30 CKD children developed LVH.^25^ Chavers et al. demonstrated that 92% of the patients presented some cardiovascular risk factors (63% hypertension, 38% anemia, 11% BMI >94th percentile, 63% serum phosphorus >5.5 mg/dL and 55% calcium-phosphorus product ≥55 mg (2)/dL (2)). A diagnosis of cardiac disease was reported in 24% of patients: LVH—17%, congestive heart failure/pulmonary edema—8%, cardiomyopathy—2% and decreased left ventricular function—2% of children.^17^

In the CKD study, the features associated with elevated BP included: black race, shorter duration of CKD, the absence of antihypertensive medications and elevated serum potassium.^19^ The risk factors of hypertension development comprise also an acquired cause of CKD, hypertension before dialysis, poor dialysis efficacy, erythropoietin administration, shorter dialysis duration and lower mean hemoglobin and calcium levels.^2^^,^^7^^,^^20^ Schaefer concluded that fluid overload, activation of the renin-angiotensin system, sympathetic hyperactivation, endothelial dysfunction and chronic hyperparathyroidism were contributing to CKD-associated hypertension.^7^ In the current study, no correlation with any of the analyzed biochemical (vitamin D, serum albumin) and dialysis adequacy parameters was demonstrated, which confirms the complexity of hypertension pathophysiology in this exceptional group of children.

Previous studies showed that patients with uncontrolled hypertension were younger and had shorter CKD 5 stage duration.^2^^,^^7^^,^^20^ Our study showed that hypertension was equally prevalent in younger and older children. This could be an effect of relatively small number of patient and different racial distribution in the population (all Caucasians).

In the most extensive analysis by Hallbach at al., significant differences in BP control by dialysis modality and disease etiology were assessed and the highest percentage of uncontrolled hypertension was found in hemodialyzed patients and patients with glomerular diseases.^20^ The present analysis some clinical entities (glomerulonephritis, hemolytic uremic syndrome and polycystic kidneys) might be risk factors for hypertension in dialyzed children. The present study failed to confirm the effect of dialysis method and efficacy, and anemia treatment on the blood pressure values.

With respect to fluid overload as a risk factor for high blood pressure, both direct and indirect measurements were applied. In the 2003 cohort, relative fluid overload was assessed by hemoglobin concentration or total protein/albumin concentration.^1^ Some authors confirmed benefits from strict fluid control in reduction of antihypertensive treatment.^26^ In the study of Vandervoorde et al., hypertension was related to hypervolemia.^18^

The present study has some strengths and limitations. Firstly, a cross-sectional analysis was designed to compare the populations of dialyzed children after a decade. The important point of this analysis was the participation of all (but one) the same pediatric dialysis centers in Poland. There was no bias in changing the treatment policy due to change of attending physicians. All the children in the study were of the Caucasian origin, which allowed to use the recently published national growth and blood pressure population charts.

On the other hand, the cross-sectional design of the study provided reliable data on the hypertension prevalence (as it was aimed), but a longitudinal observation of the children cohort could have given more data on risk factors associated with the RRT procedures.

Relatively small number of patients in the 2013 cohort might be a weak point of the paper. The number of dialyzed children decreased within this period, which may be noted as a success of pediatric nephrology and transplantation in Poland. During the last decade, the overall number of dialyzed children in Poland decreased of 40% due to more efficient diagnosis and nephroprotection (data from Polish Registry of Dialyzed Children—unpublished) and higher rate of successful transplantations. To overcome this weakness, authors combined two cohorts of patients to obtain statistical significance in the multiple regression analysis.

From the methodological point of view, it should be pointed that the anthropometric and blood pressure measurements were not standardized for every center and hypertension was not confirmed by ABPM. Nevertheless, according to the 4th Report, casual blood pressure measurement is an accepted method of diagnosing hypertension. In 2003, there was no adequate equipment for ABPM in Polish dialysis units.^27^ Authors intended to use this method in the cohort of 2013, but the comparison was not possible, and thus, the data were not presented.

Summarizing the results of this study, it should be emphasized that despite the availability of modern diagnostic and treatment methods, in chronically dialyzed children in Poland, hypertension is highly prevalent, partially underdiagnosed and in most cases undertreated.

A significant improvement in this field was expected, but after comparing the data from 2003 and 2013, no progress was made during this period, despite the success in reduction of number of dialyzed children.

## References

[1] Tkaczyk M, Nowicki M, Bałasz-Chmielewska I, et al Hypertension in dialysed children: The prevalence and theraputic approcach in Poland - a nationwide survey. Nephrol Dial Transplant. 2006;21:736–742. 16303782 10.1093/ndt/gfi280

[2] Chavers BM, Solid CA, Daniels FX, et al Hypertension in pediatric long-term hemodialysis patients in the United States. Clin J Am Soc Nephrol. 2009;4:1363–1369. 19556378 10.2215/CJN.01440209 PMC2723970

[3] Mitsnefes MSD. Hypertension in pediatric patients on long-term dialysis: A report of the North American Pediatric Renal Transplant Cooperative Study (NAPRTCS). Am J Kidney Dis. 2005;45:309–315. 15685509 10.1053/j.ajkd.2004.11.006

[4] Van Buren PN, Inrig JK. Hypertension and hemodialysis: Pathophysiology and outcomes in adult and pediatric populations. Pediatr Nephrol. 2012;27:339–350. 21286758 10.1007/s00467-011-1775-3 PMC3204338

[5] Chavers BM, Li S, Collins AJ, Herzog CA. Cardiovascular disease in pediatric chronic dialysis patients. Kidney Int. 2002;62:648–653. 12110030 10.1046/j.1523-1755.2002.00472.x

[6] Parekh RS, Carroll CE, Wolfe RA, Port FK. Cardiovascular mortality in children and young adults with end-stage kidney disease. J Pediatr. 2002;141:191–197. 12183713 10.1067/mpd.2002.125910

[7] Hadtstein C, Schaefer F. Hypertension in children with chronic kidney disease: pathophysiology and management. Pediatr Nephrol. 2008;23:363–371. 17990006 10.1007/s00467-007-0643-7 PMC2214827

[8] Agarwal R, Nissenson AR, Batlle D, Coyne DW, Trout JR, Warnock MD. Prevalence, treatment, and control of hypertension in chronic hemodialysis patients in the United States. Am J Med. 2003;115:291–297. 12967694 10.1016/s0002-9343(03)00366-8

[9] Mitsnefes M. Cardiovascular disease in children with chronic kidney disease. Adv Chronic Kidney Dis. 2005;12:397–405. 16198279 10.1053/j.ackd.2005.07.005

[10] Hooper DK, Williams JC, Carle AC, et al The quality of cardiovascular disease care for adolescents with kidney disease: A Midwest Pediatric Nephrology Consortium study. Pediatr Nephrol. 2013;28:939–949. 23417277 10.1007/s00467-013-2419-6 PMC3637925

[11] Canella G, Paoletti E, Ravera G, et al Inadequate diagnosis and therapy of arterial hypertension as causes of left ventricular hypertrophy in uremic dialysis patients. Kidney Int. 2000;58:260–268. 10886571 10.1046/j.1523-1755.2000.00161.x

[12] Kulaga Z, Litwin M, Grajda A, *et al* Oscillometric blood pressure percentiles for Polish normal-weight school-aged children and adolescents. J Hypertens. 2012;30:1942–1954. 22828086 10.1097/HJH.0b013e328356abad

[13] National High Blood Pressure Education Program Working Group on High Blood Pressure in Children and Adolescents. The fourth report on the diagnosis, evaluation, and treatment of high blood pressure in children and adolescents. Pediatrics. 2004;114:555–576. 15286277

[14] Kulaga Z, Litwin M, Tkaczyk M, et al Polish 2010 growth references for school-aged children and adolescents. Eur J Pediatr. 2011;170:599–609. 20972688 10.1007/s00431-010-1329-x PMC3078309

[15] Kulaga Z, Grajda A, Gurzkowska B, et al Polish 2012 growth references for preschool children. Eur J Pediatr. 2013;172:753–761. 23371392 10.1007/s00431-013-1954-2 PMC3663205

[16] Kramer AM, van Stralen KJ, Jager KJ, et al Demographics of blood pressure and hypertension in children on renal replacement therapy in Europe. Kidney Int. 2011;80:1092–1098. 21814180 10.1038/ki.2011.232

[17] Chavers BM, Solid CA, Sinaiko A, et al Diagnosis of cardiac disease in pediatric end-stage renal disease. Nephrol Dial Transplant. 2011;26:1640–1645. 20861193 10.1093/ndt/gfq591 PMC3145383

[18] Vandevoorde RG, Barletta GM, Chand DH, et al Blood pressure control in pediatric hemodialysis: The Midwest Pediatric Nephrology Consortium Study. Pediatr Nephrol. 2007;22:547–553. 17115195 10.1007/s00467-006-0341-x

[19] Flynn JT, Mitsnefes M, Pierce C, *et al* Blood pressure in children with chronic kidney disease: A report from the Chronic Kidney Disease in Children Study. Hypertension. 2008;52:631–637. 18725579 10.1161/HYPERTENSIONAHA.108.110635 PMC3136362

[20] Halbach SM, Martz K, Mattoo T, Flynn J. Predictors of blood pressure and its control in pediatric patients receiving dialysis. J Pediatr. 2012;160:621–625. 22056352 10.1016/j.jpeds.2011.09.046 PMC3409690

[21] Mitsnefes M. Cardiovascular complications of pediatric chronic kidney disease. Pediatr Nephrol. 2008; 23:27–39. 17120060 10.1007/s00467-006-0359-0 PMC2100430

[22] Tjaden LA, Vogelzang J, Jager KJ, et al Long-term quality of life and social outcome of childhood end-stage renal disease. J Pediatr. 2014;165:336–342. 24837864 10.1016/j.jpeds.2014.04.013

[23] Mitsnefes MM, Laskin BL, Dahhou M, Zhang X, Foster BJ. Mortality risk among children initially treated with dialysis for end-stage kidney disease, 1990-2010. JAMA. 2013;309:1921–1929. 23645144 10.1001/jama.2013.4208 PMC3712648

[24] Bakkaloglu SA, Borzych D, Soo HI, et al Cardiac geometry in children receiving chronic peritoneal dialysis: Findings from the International Pediatric Peritoneal Dialysis Network (IPPN) registry. Clin J Am Soc Nephrol. 2011;6:1926–1933. 21737855 10.2215/CJN.05990710 PMC3359542

[25] Drozdz D, Kordon Z, Pietrzyk JA, Drozdz M, Rudziński A, Zachwieja K. The assessment of heart function in children with chronic kidney disease (CKD). Pol Merkur Lekarski. 2008;24:98–100. 18924516

[26] Candan C, Sever L, Civilibal M, Caliskan S, Arisoy N. Blood volume monitoring to adjust dry weight in hypertensive pediatric hemodialysis patients. Pediatr Nephrol. 2009;24:581–587. 18781335 10.1007/s00467-008-0985-9

[27] Chaudhuri A, Sutherland SM, Begin B, *et al* Role of twenty-four-hour ambulatory blood pressure monitoring in children on dialysis. Clin J Am Soc Nephrol. 2011;6:870–876. 21273374 10.2215/CJN.07960910 PMC3069381

